# Seasonal trends and short-term association between meteorological factors and diarrheal diseases among children under five in Tanzania

**DOI:** 10.1371/journal.pone.0357174

**Published:** 2026-08-28

**Authors:** Gerald P. Mwing’a, Mariana Shayo, Patrick Kimu, Melkiory Beti, Boaz Wadugu, Lameck Pashet, Masoud A. Juma, Laura Shirima, Davis Kuchaka, Happiness Kumburu, Lucas Matemba, Paul E. Kazyoba, Calvin Sindato, Angelina M. Lutambi, Florida Muro, Blandina T. Mmbaga, Michael Alifrangis, Frank M. Aarestrup, Tolbert Sonda

**Affiliations:** 1 Department of Epidemiology and Biostatistics, School of Public Health, KCMC University, Moshi, Tanzania; 2 National Institute for Medical Research, Dodoma, Tanzania; 3 Kilimanjaro Clinical Research Institute, Moshi, Tanzania; 4 Department of Biological and Pre-Clinical Studies, Muhimbili University of Health and Allied Sciences, Dar es Salaam, Tanzania; 5 Kishapu District Council, Shinyanga, Tanzania; 6 The State University of Zanzibar, Zanzibar, Tanzania; 7 Department of Paediatric and Child Health, School of Medicine, KCMC University, Moshi, Tanzania; 8 Centre for Translational Medicine and Parasitology, Department of Immunology and Microbiology, University of Copenhagen, Department of Infectious Diseases, Copenhagen University Hospital (Rigshospitalet), Copenhagen, Denmark; 9 Technical University of Denmark, Lundtofte, Denmark; Clinton Health Access Initiative, UNITED STATES OF AMERICA

## Abstract

Childhood Diarrhea remains one of the leading causes of morbidity and mortality among children under five in Tanzania. Although meteorological factors are known to influence childhood diarrhea, evidence on their short-term associations across different regions of Tanzania remains limited. This study aimed to determine the seasonal prevalence of childhood diarrhea and its short-term associations with meteorological factors among children under five years in Tanzania between 1 May 2023 and 30 April 2024.

**Methodology:**

This retrospective analysis of repeated cross-sectional surveillance data included children under five years attending 10 selected healthcare facilities across seven regions of Tanzania between 1 May 2023 and 30 April 2024. Routinely collected health data from the Seq-Tanzania project, stored in the District Health Information System 2 (DHIS2), were linked with meteorological data obtained from the Tanzania Meteorological Authority (TMA). A multilevel mixed-effects Poisson regression model with a log link and robust standard errors was fitted to identify the short-term associations between meteorological and sociodemographic factors and childhood diarrhea.

**Results:**

A total of 898 children under five were included in the analysis, with a median age (IQR) of 13.9 (8.8–25.3) months. The prevalence of childhood diarrhea was highest during the dry season (58%) in regions experiencing unimodal rainfall patterns and during the wet season (41%) in the regions experiencing bimodal rainfall patterns. In the final multilevel mixed-effects Poisson regression model, higher average monthly temperature, increased total monthly rainfall, younger child age, and maternal primary education were significantly associated with the prevalence of childhood diarrhea.

**Conclusion:**

Average monthly temperature and total monthly rainfall were significantly associated with the prevalence of childhood diarrhea across regions with both unimodal and bimodal rainfall patterns in Tanzania. These findings highlight the importance of integrating meteorological information into diarrheal disease surveillance and public health planning to support timely interventions aimed at reducing childhood diarrhea.

## Background

Diarrheal diseases remain a major public health challenge and are the second leading cause of childhood mortality worldwide, accounting for about 1.7 billion cases and 525,000 deaths in children under five, annually [1]. The disease contributes to approximately 9% of all under-five mortality worldwide, with nearly 90% occurring in Sub-Saharan Africa [2]. In Tanzania, diarrhea accounts for approximately 9% of all under-five mortality, positioning the country at number 10 among the 15 nations with the highest burden of diarrhea-related fatalities among children under five [3]. Children under five are particularly vulnerable because of their immature immune systems, increased exposure to environmental contaminants, and limited ability to protect themselves from adverse environmental conditions [4]. They are also less knowledgeable about the health effects of climate change, with less ability to remove themselves from the threat [4].

Diarrhea diseases are among the climate-sensitive diseases caused by bacterial, viral, and parasitic pathogens, which are transmitted primarily through the fecal–oral route [5]. Their transmission dynamics are influenced by meteorological factors, including humidity, rainfall and temperature [6,7]. Increased temperatures mostly enhance the proliferation of bacteria in water and food, prolong pathogen survival, and elevate the probability of an outbreak [8]. On the other hand, floods and heavy rainfall may overwhelm sanitation infrastructures and contaminate drinking water sources, while drought reduces water availability and compromises hygiene practices [9]. Climatic conditions such as warmer and humid conditions may also indirectly facilitate by favouring vectors such as flies and other insects that contribute to the spread of diarrheal pathogens.

Tanzania experiences two distinct rainfall regimes: bimodal and unimodal rainfall patterns [10,11]. Bimodal regions receive two rainy seasons annually, from March to May, and from November to December, separated by the dry seasons between June and October, and January and February. In contrast, unimodal regions experience one rainy season from November to April, followed by a prolonged dry season from May to October [11]. These climatic differences may influence the seasonal distribution of childhood diarrhea across regions.

Evidence from East Africa and other sub-Saharan African countries demonstrates that climatic variability plays an important role in the epidemiology of childhood diarrhea. Studies from Ethiopia have reported that increasing temperature and rainfall are associated with higher childhood diarrhea incidence, although the magnitude of these associations varies across ecological settings and seasons [7,12]. Similarly, studies conducted in Kenya have shown that climatic variability, together with socioeconomic factors and access to water, sanitation and hygiene (WASH) services, significantly influences childhood diarrhea [13]. Across sub-Saharan Africa, disparities in water supply, sanitation infrastructure, and hygiene practices further modify the relationship between climate variability and childhood diarrhea, particularly during periods of heavy rainfall and drought [14,15].

Previous studies have shown that seasonal variation, rainfall, and temperature influence childhood diarrhea, although the magnitude and direction of these associations vary across countries because of differences in geographical, climatic, socioeconomic, and hygiene conditions [16–21]. Despite this evidence, studies examining the seasonal prevalence and short-term associations between meteorological factors and childhood diarrhea across Tanzania’s diverse climatic zones remain limited. Understanding these relationships is important for strengthening disease surveillance, improving preparedness, and guiding timely public health interventions. Therefore, this study aimed to determine the seasonal prevalence of childhood diarrhea and its short-term associations with meteorological factors among children under five years in Tanzania.

## Materials and methods

### Data sources

The dataset comprised routinely collected surveillance data from healthcare facilities participating in the Seq-Tanzania project. These data were prospectively collected during routine healthcare encounters between 1 May 2023 and 30 April 2024 and entered into the District Health Information System (DHIS2) managed by the Kilimanjaro Clinical Research Institute (KCRI). For the present study, the records were retrospectively extracted from DHIS2 on 3 June 2024 for secondary analysis. Daily rainfall (mm) and daily mean temperature (°C) were obtained from the Tanzania Meteorological Authority (TMA). Meteorological data, including daily average rainfall (mm) and daily mean temperature (^0^C), were obtained from the Tanzania Meteorological Authority (TMA) for weather stations corresponding to each study region. Daily observations were aggregated into monthly average temperature and total monthly rainfall and linked to the health surveillance data using the healthcare facility location and month of patient presentation to evaluate short-term associations between weather conditions and childhood diarrheal diseases. Permission to access and analyse the surveillance data was obtained from the Seq-Tanzania project management and the Kilimanjaro Clinical Research Institute. The corresponding author was an authorised member of the Seq-Tanzania Project team and had permission to access the de-identified data for research purposes.

### Study design and settings

The study was a retrospective analysis of a repeated cross-sectional study collected through the Seq-Tanzania project between 1 May 2023 and 30 April 2024. The Seq-Tanzania project integrated routinely collected health data from children under five years attending selected healthcare facilities with corresponding meteorological data obtained from the Tanzania Meteorological Authority (TMA) before storing the linked dataset in the District Health Information System 2 (DHIS2). The present study involved a secondary analysis of these linked surveillance data. The study was implemented in the seven regions of Tanzania, namely Kaskazini Unguja, Mjini Magharibi, Mwanza, Tanga, Dodoma, Tabora, and Mbeya, representing both unimodal and bimodal rainfall patterns. Ten healthcare facilities participating in the Seq-Tanzania surveillance project were purposively selected based on their geographical distribution, patient volume, laboratory capacity for molecular analyses, and ability to provide continuous surveillance data throughout the study period. These facilities were distributed across regions to capture the diversity of climatic conditions across Tanzania and facilitate the assessment of seasonal variations in childhood diarrheal diseases.

### Study population

**Fig 1:**
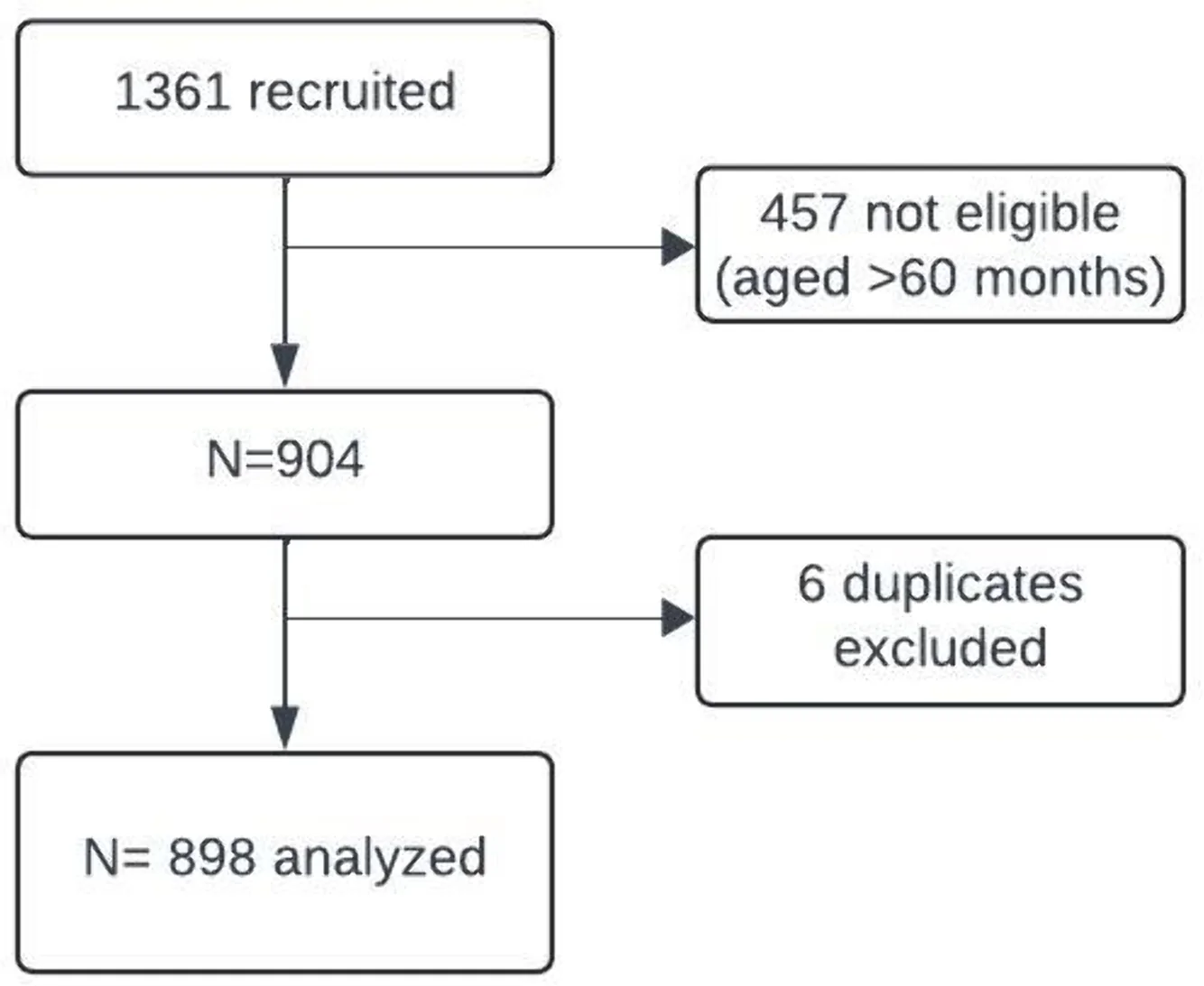
The flow chart for the selection of the study participants.

The study used data from the District Health Information System (DHIS2), which was extracted from the system on 03 June 2024. A total of 1361 participants were extracted from the system; of them, 457, who were aged greater than 60 months, and those with duplicate information (n = 6) were excluded. Therefore, each observation included in this study represents a cross-sectional assessment recorded at the time of the healthcare visit. The present analysis aggregated these repeated cross-sectional observations over 12 months to assess seasonal trends and short-term associations between climatic variables and childhood diarrhea. Thus, the analysis took place on a total of 898 children who met the inclusion criteria (Fig 1).

### Study variables

#### Dependent (outcome) variable.

The outcome variable was childhood diarrhea, assessed during routine healthcare visits using the standardised Seq-Tanzania surveillance tool. Trained healthcare workers interviewed parents or legal guardians and recorded diarrhea status (Yes/No) according to the World Health Organisation (WHO) case definition, defined as the passage of three or more loose or liquid stools within 24 hours [5]. The information was subsequently entered into the DHIS2 surveillance database for routine monitoring.

#### Independent variables.

Independent variables were classified based on the multilevel framework into individual-level and community-level (meteorological) variables based on biological plausibility and previous literature [22–24]. Individual-level variables included child age (months), sex, maternal education, mother’s hand washing practices, food preservation, exclusive breastfeeding, drinking water sources, latrine type, and type of residence. Drinking water sources were categorised as improved (covered hand-dug wells, Municipality water supply, private water tap/pump, public water tap/pump, protected home water, and harvesting rainwater) or unimproved (uncovered hand-dug wells, ponds, rivers/streams, and dams) according to established classification [25,26]. Residences were classified as formal or informal settlements based on the availability of basic infrastructure and services [27]. Community-level variables comprised monthly average temperature (°C) and total monthly rainfall (mm), obtained from regional meteorological stations through the Tanzania Meteorological Authority (TMA).

### Data management and statistical analysis

Data were exported from the DHIS2 system into Microsoft Excel for quick view, cleaning and preliminary visualisation before being analysed using STATA version 17 software (StataCorp LLC, College Station, TX, USA). Descriptive statistics were applied to summarise participant characteristics, with Pearson’s chi-square test being used to compare the distributions of childhood diarrhea across categories of independent variables. Factors associated with childhood diarrhea were assessed using a multilevel mixed-effects Poisson regression model with a log link function and robust standard errors. Because childhood diarrhea was a common outcome (>10%), adjusted prevalence ratios (APR) were estimated instead of odds ratios, as prevalence ratios provide more accurate and interpretable measures of association for common outcomes. A healthcare facility was included as a random intercept to account for clustering of children within facilities. Fixed effects included child age (months), sex, maternal education level, breastfeeding status, drinking water source, monthly average temperature, and total monthly rainfall. Results were presented as adjusted prevalence ratios (APR) with 95% confidence intervals (CI).

Model building followed a hierarchical approach. Model I was an empty (null) model containing only facility-level random effects. Model II included meteorological variables, Model III included individual-level variables, and Model IV included both meteorological and individual-level variables. Multicollinearity was assessed using the variance inflation factor (VIF), with values <10 indicating no evidence of problematic multicollinearity. The missing data were handled using a complete case analysis [28]. Model performance was evaluated using the Akaike Information Criterion (AIC), log-likelihood, deviance, intraclass correlation coefficient (ICC), and median prevalence ratio (MPR) [29]. The model with the lowest AIC and deviance and the highest log-likelihood was considered the best-fitting model. Statistical significance was assessed using a two-sided p-value <0.05.

### Ethical approval

This study was a secondary analysis of routinely collected surveillance data from the Seq-Tanzania project. Ethical approval for the Seq-Tanzania project was obtained from the Tanzania National Institute for Medical Research’s Medical Research Coordinating Committee (Ref. No. NIMR/HQ/R.8a/Vol.IX/3859). During the original data collection, written informed consent was obtained from adult participants and from the parents or legal guardians of children, while assent was obtained from children where applicable. The present study analysed de-identified data and did not involve direct participant contact or collection of additional information. Permission to access and analyse the data was obtained from the Seq-Tanzania project and the relevant institutional authorities.

## Results

### Characteristics of study participants

A total of 898 children under five were included in the analysis. The median age (IQR) was 13.9 (8.8–25.3) months, with 30.4% aged 6–11 months. More than half (57%) were male, and 90.9% had received the rotavirus vaccine. Among infants aged <6 months with available breastfeeding information, 62.9% were not exclusively breastfed for six months. Most households (90.6%) used improved drinking water sources, and 36.5% of mothers had completed primary education. During the study period, the mean monthly temperature was 24.3°C (SD ± 2.7; range: 18.5–31.2°C), while the mean total monthly rainfall was 110.4 mm (SD ± 145.2; range: 0–775.9 mm) (Table 1).

**Table 1:** Characteristics of the study participants (N = 898).

Characteristics	Frequency (n)	Per cent (%)
Median age (IQR) = 13.9(8.8–25.3) months		
**Age**		
<6 months	95	10.6
6–11 months	273	30.4
12-23 months	268	29.8
24-59 months	262	29.2
**Sex**		
Female	386	43
Male	512	57
**Mother’s education level** ^**a**^		
Primary education	316	36.5
Secondary education	309	35.6
College and higher	242	27.9
**Region**		
Dodoma	195	21.7
Kaskazini Unguja	47	5.2
Mbeya	196	21.8
Mjini Magharibi	52	5.8
Mwanza	181	20.2
Tabora	46	5.1
Tanga	181	20.2
**Residence** ^**b**^		
Informal settlements	123	13.8
Formal settlements	766	86.2
**Rotavirus vaccine status**		
Not vaccinated	82	9.1
Vaccinated	816	90.9
**Infants aged <6 months ebf for 6 months** ^**c**^		
No	44	62.9
Yes	26	37.1
**Drinking water sources** ^**d**^		
Unimproved	80	9.4
Improved	773	90.6
**Drinking water storage** ^**e**^		
Unsealed container	5	0.6
Sealed container	817	99.4
**Latrine type** ^**f**^		
Pit latrine	159	19.1
Flush latrine	674	80.9
Average monthly temperature (SD) = 24.3 ^0^C (±2.7), min. of 18.5°C and max. of 31.2°C		
Average total monthly rainfall (SD) = 110.4 mm (±145.2) ranging from 0 to 775.9 mm		

^a^(n = 867), ^b^ (n = 889), ^c^ (n = 70), ^d^ (n = 853), ^e^ (n = 822), ^f^ (n = 833), ebf = exclusive breastfeeding.

### Prevalence of childhood diarrhea by participant characteristics

Overall, 622 of the 898 children under five (69.3%) had childhood diarrhea during the study period. The prevalence of childhood diarrhea differed significantly across age groups, maternal education levels, and drinking water sources (p < 0.05). Children aged 6–11 months, those whose mothers had college or higher education, and those from households using unimproved drinking water sources had the highest prevalence of childhood diarrhea (Table 2).

**Table 2:** Prevalence of childhood diarrhea by participant characteristics (N = 898).

Variables	Diarrhea in children under five		P-value
	Yes n (%)	No n (%)	
**Total**	622(69.3)	276(30.7)	
**Age of a child**			
<6 months	66(69.5)	29(30.5)	<0.001
6–11 months	213(78.0)	60(22.0)	
12-23 months	184(68.7)	84(31.3)	
24-59 months	159(60.7)	103(39.3)	
**Sex**			
Female	270(69.9)	116(30.1)	0.700
Male	352(68.8)	160(31.3)	
**Mother’s education level** ^**a**^			
Primary education	197(62.3)	119(37.7)	0.002
Secondary education	217(70.2)	92(29.8)	
College and higher	184(76.0)	58(24.0)	
**Residence** ^**b**^			
Informal settlement	87(70.7)	36(29.3)	0.709
Formal settlement	529(69.1)	237(30.9)	
**Rotavirus vaccine**			
No	64(78.1)	18(21.9)	0.071
Yes	558(68.4)	258(31.6)	
**Infant aged <6 months EBF for six months** ^**c**^			
Yes	23(88.5)	3(11.5)	0.083
No	31(70.5)	13(29.5)	
**Water sources** ^**d**^			
Unimproved	64(80)	16(20)	0.023
Improved	523(67.7)	250(32.3)	

^a^(n=867), ^b^ (n=889) ^c^ (n=70), ^d^ (n=853).

### Seasonal prevalence of childhood diarrhea by rainfall pattern

**Fig 2:**
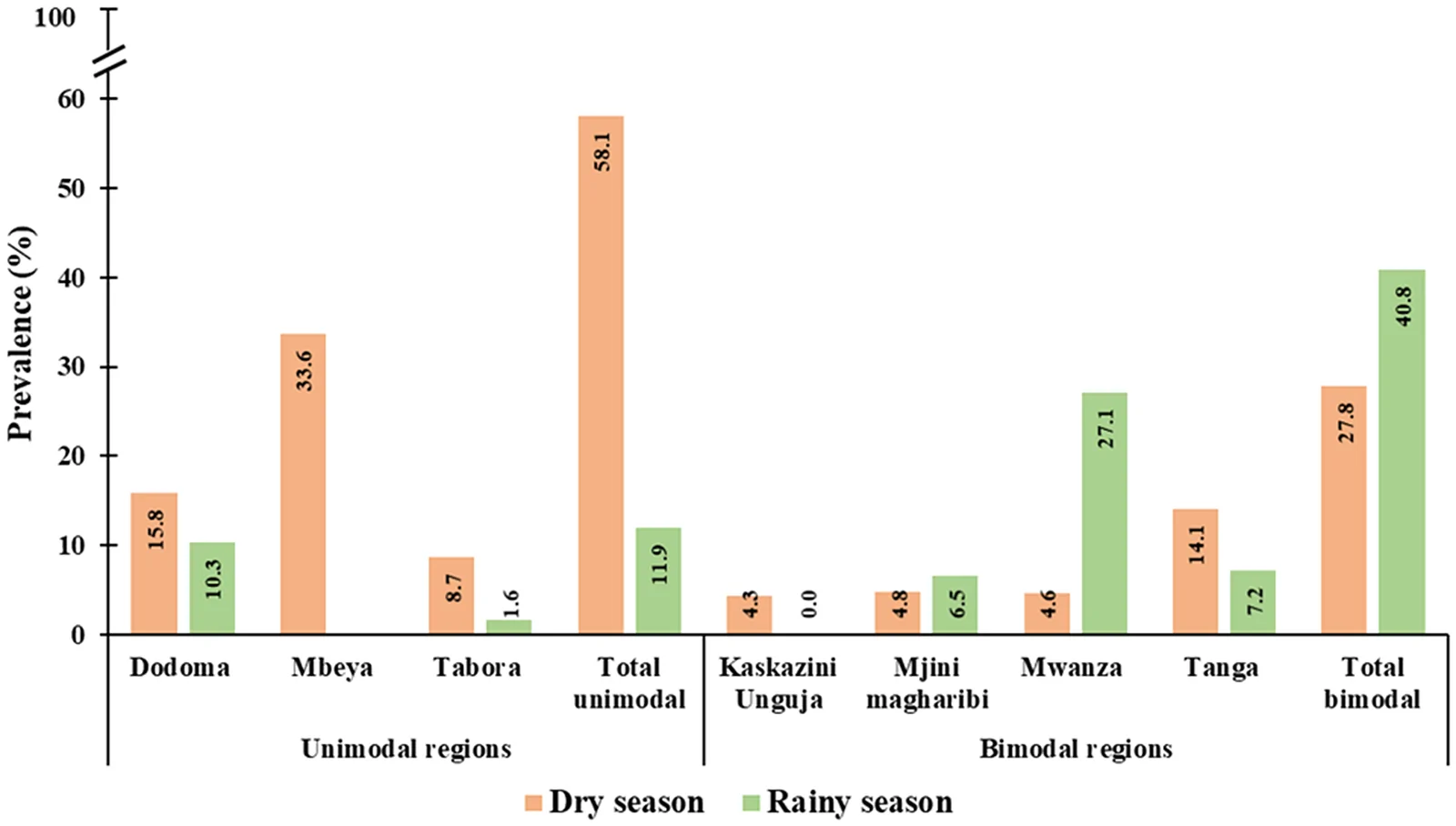
Seasonal prevalence of childhood diarrhea in regions with unimodal and bimodal rainfall patterns.

Among the 898 children, 437 (48.7%) resided in regions with unimodal rainfall patterns and 461 (51.3%) in regions with bimodal rainfall patterns. In the unimodal regions, childhood diarrhea was more prevalent during the dry season (58%; 95% CI: 53.3–62.8) than during the rainy season, with the Mbeya region contributing the highest regional prevalence (33.6%; 95% CI: 29.2–38.3). Conversely, in the bimodal regions, childhood diarrhea was highest during the rainy season (41%; 95% CI: 33.5–42.4), largely driven by the Mwanza region (27.1%; 95% CI: 23.1–31.4), followed by the Tanga and Mjini magharibi regions (approximately 7% each). No childhood diarrhea cases were recorded in Kaskazini Unguja during the rainy seasons (Fig 2).

### Seasonal trends of childhood diarrhea in relation to meteorological factors

#### Regions with unimodal rainfall patterns.

**Fig 3:**
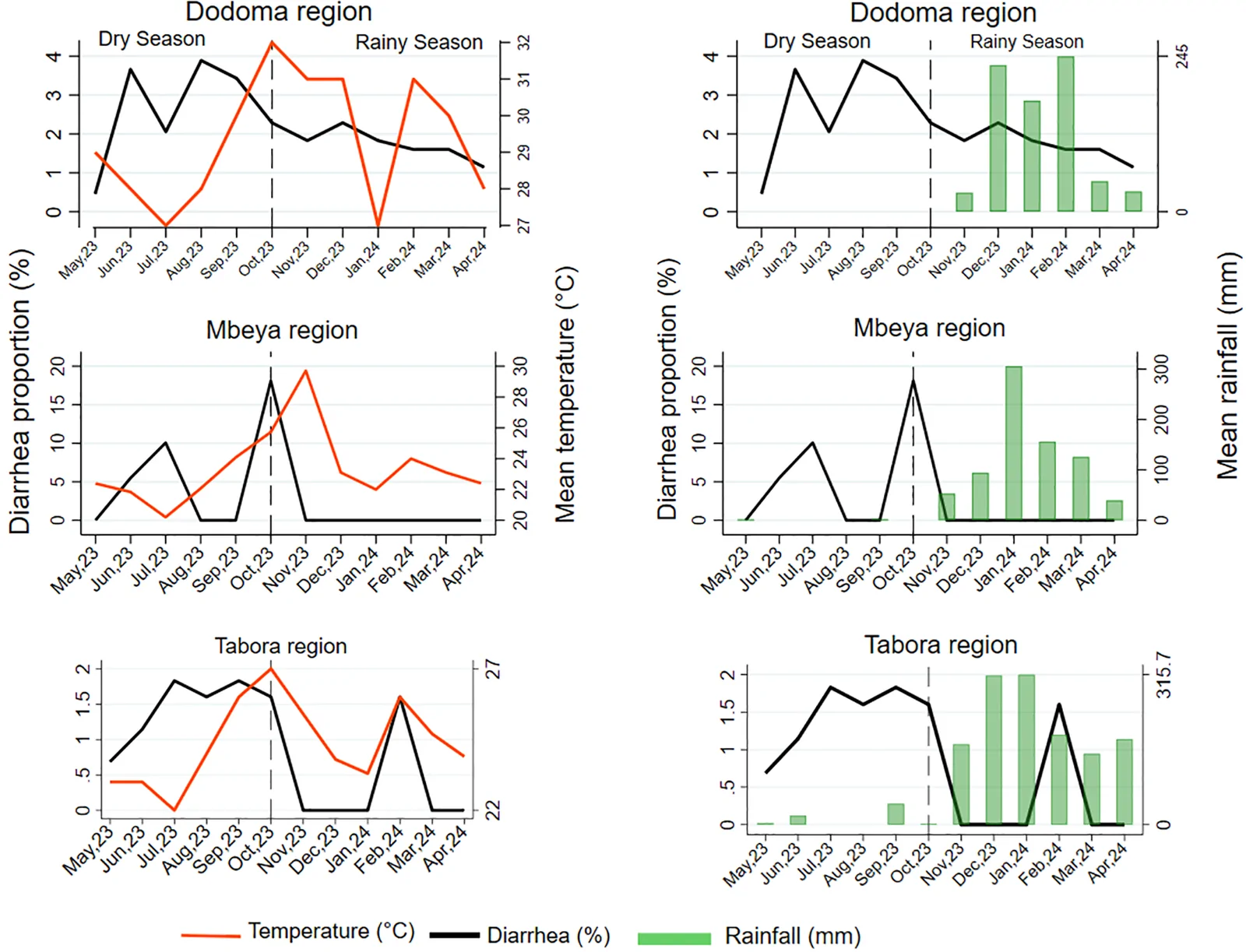
Monthly trends of childhood diarrhea in relation to average temperature and total rainfall in regions with unimodal rainfall patterns.

Fig 3 shows the monthly trends in childhood diarrhea alongside average monthly temperature and total monthly rainfall in the unimodal regions. The highest prevalence was observed in October (22%; 95% CI: 18.2–26.1%) during the dry season, predominantly in Mbeya region (18.1%; 95% CI: 14.6–22.0%), when the average monthly temperature was 26^0^C and rainfall was negligible. Higher temperatures coincided with increased childhood diarrhea prevalence, whereas diarrhea prevalence generally declined during months with increased rainfall, except for a slight increase observed in Tabora in February (Fig 3).

#### Regions with bimodal rainfall patterns.

**Fig 4:**
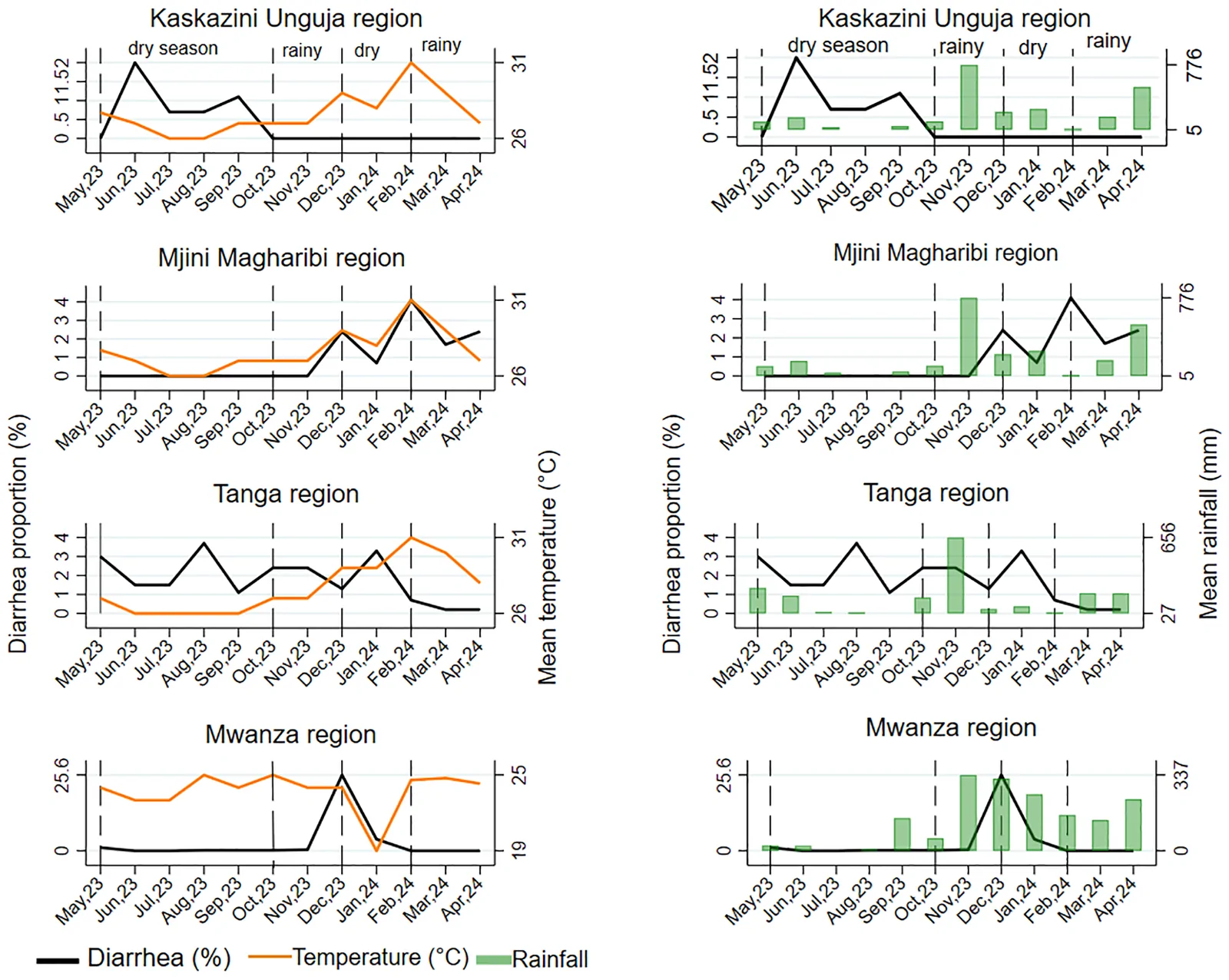
Monthly trends of childhood diarrhea in relation to average temperature and total rainfall in regions with bimodal rainfall patterns.

Fig 4 presents the monthly trends in childhood diarrhea in relation to temperature and rainfall in the bimodal regions. The highest prevalence occurred in December (29.3%; 95% CI: 25.2–33.7%) during the rainy season, with the Mwaza region contributing highest prevalence (26%; 95% CI: 21.7–29.8%) at an average monthly temperature of 24 ^0^C and 321 mm of rainfall compared to other regions. In the Mwanza region, childhood diarrhea increased from November, peaked in December, and declined in January onwards as rainfall and temperature decreased (Fig 4).

### Factors associated with childhood diarrhea

Model IV, which included both individual-level factors and meteorological variables, provided the best fit to the data and was therefore selected as the final model. After adjusting for clustering at the healthcare facility level and other covariates, total monthly rainfall, average monthly temperature, child age, and mother’s education level remained significantly associated with childhood diarrhea. Specifically, each 1 mm increase in total monthly rainfall (mm) was associated with 0.08% higher prevalence of childhood diarrhea (APR: 1.0008; 95% CI: 1.0003–1.0013), while each 1 ^0^C increase in average monthly temperature was associated with an 11% higher prevalence (APR: 1.11; 95% CI: 1.05–1.17). Subsequently, each additional month of age of a child was associated with 2% lower prevalence of childhood diarrhea (APR: 0.98; 95% CI: 0.97–0.99). Similarly, children whose mothers had a primary level of education had a 9% lower prevalence of childhood diarrhea than those whose mothers had college or higher education levels (APR: 0.91; 95% CI: 0.85–0.98). The selection of Model IV was supported by its lowest AIC (1409.22) and deviance (1393.21), together with the highest log-likelihood (−696.61) among the four models. Furthermore, the null model ICC of 5.5% indicated modest clustering of childhood diarrhea within healthcare facilities, supporting the use of a multilevel modelling approach (Table 3).

**Table 3:** Multilevel mixed-effects Poisson regression analysis of meteorological and individual-level factors associated with childhood diarrhea in Tanzania (N = 898).

Characteristics	Null Model (Model I)	MODEL II	MODEL III	MODEL IV
		Meteorological factors	Individual-level factors	Meteorological and Individual-level factors
		APR (95% CI)	APR (95% CI)	APR (95% CI)
**Fixed effects**				
**Seasonal variables**				
Rainfall		1.001(1.0004 - 1.0013) **		1.0008(1.0003 - 1.0013) **
Temperature		1.11(1.06 - 1.18) **		1.11(1.05 - 1.17) **
**Age in months**			0.98(0.97 - 0.99) **	0.98(0.97 - 0.99) **
**Sex**				
Female			1	1
Male			0.95(0.91 - 1.01)	0.96(0.91 −1.01)
**Mother’s education level**				
College/higher			1	1
Primary			0.86(0.77 - 0.97) **	0.91(0.85 - 0.98) *
Secondary			0.94(0.86 - 1.04)	0.92(0.84 - 1.00)
**Rotavirus vaccine**				
Yes			1	
No			1.20(1.01 - 1.42) **	
**Child breastfeeding status**				
≥ 6 months			1	1
< 6 months			0.85(0.72 - 1.01)	0.85(0.71 - 1.02)
**Water sources**				
Improved			1	1
Unimproved			1.11(1.02 - 1.22) *	0.98(0.88 - 1.08)
ICC	5.5%	10.71%	2.15%	7.66%
AIC	1691.44	1663.559	1433.49	1409.22
Variability between	0.054	0.12	0.022	0.083
Variability within	1	1	1	1
MPR	1.25 (1.11, 1.34)	1.39 (1.16, 1.56)	1.15 (1.0, 1.24)	1.32 (1.08, 1.47)
**Model comparison**				
Likelihood	−843.79	−827.7793	−708.74	−696.61
Deviance	1687.58	1655.5586	1417.48	1393.21

* = P < 0.05, ** = P < 0.01. ICC: Intraclass correlation coefficient, AIC: Akaike information criteria, APR: Adjusted Prevalence Ratio, MPR: Median prevalence ratio.

Model I: Empty/null model.

Model II: Adjusted for meteorological factors.

Model III: Adjusted for individual-level factors.

Model IV: Adjusted for meteorological and individual-level factors.

## Discussion

This study examined the seasonal prevalence of childhood diarrhea and its short-term association with meteorological factors among children under five years in Tanzania. The findings demonstrated clear seasonal variation in childhood diarrhea across regions with unimodal and bimodal rainfall patterns. In the unimodal regions, the highest prevalence was observed during the dry season (May 2023 – October 2023), particularly in October in the Mbeya region, whereas in the bimodal regions, childhood diarrhea peaked during the rainy season, with the highest prevalence recorded in Mwanza in December. These seasonal differences are likely influenced by regional climatic conditions together with variations in water availability, sanitation, hygiene practices, and healthcare access. For example, the semi-arid conditions in Dodoma and Tabora may contribute to water scarcity and poor hygiene during the dry season, while rapid urbanisation, inadequate sanitation infrastructure, and flooding may increase environmental contamination in Mwanza during the rainy season [16,17,29–35]. Similar seasonal patterns have been reported in other African settings, although the timing and magnitude of seasonal peaks vary according to local climatic, environmental, and socioeconomic conditions [7,31,36,37].

In regions that receive rainy seasons twice per year (bimodal), the prevalence of childhood diarrhea was found to be high during the rainy seasons from March-May, and November –December, with the highest prevalence observed in December, by which the Mwanza region contributes the highest percentage of diarrhea than other bimodal regions. This could be due to high congestion, presence of unplanned settlements with poor management of sewage system [32,33], and inadequate waste disposal system [22]. Further, in Tanzania, during the rainy seasons, most of the households in towns tend to release wastewater to the flowing flood water channels, which in turn contaminate the environment, and when it comes into contact with children below 5 years old who live at homes playing around such an environment might carry germs that may infect them [34,35]. Generally, for the unimodal regions, diarrhea was higher in October, which is the period when the dry season ends, and for the bimodal regions, specifically the Mwanza region, diarrhea started to increase from November and reached the peak in December, which is the period when the rainy season starts. This transition period between the end and the start of rainy seasons has been associated with an increased diarrhea episode in children under five and has also been reported by several studies in Africa [38–40], though months of occurrence may differ due to differences in geographical conditions between countries, hygiene practices, water sources, and other socio-demographic factors. These findings emphasise the need for target interventions to reduce under-five diarrhea prevalence during the dry season in areas situated in the unimodal regions, and the rainy seasons in areas situated in the bimodal regions, particularly the Mwanza region. Special attention should focus on intervention in the regions that are mostly affected by seasonal variations that may help predict and set appropriate measures before diarrhea incidences.

Our findings further showed that higher average monthly temperature (^0^C) and total monthly rainfall were independently associated with increased prevalence of childhood diarrhea. The positive association with temperature is consistent with previous studies demonstrating that warmer conditions enhance the growth and survival of enteric pathogens in food and water and may reduce water availability for hygiene during dry periods [23,41–43]. High temperatures, particularly during the dry seasons, have the potential to extend the survival of pathogens, particularly bacteria and parasites in their zoonotic host, which facilitates the prolonged transmission of the pathogen [44,45]. Warmer conditions cause sequential changes in human behaviour, including increased water intake, drinking of unimproved water sources, and reduced hygiene standards due to lack of water [46,47]. Likewise, increased rainfall may contaminate drinking water sources through flooding and surface runoff, thereby increasing exposure to diarrheal pathogens [48–50]. Similar associations have been reported in Ecuador, India, China, and several African countries, although inconsistent findings across studies suggest that the effects of rainfall are context-specific and influenced by geographical, climatic, sanitation, and socioeconomic differences [14,41,51–53]. These findings highlight the need for strengthening water quality management, sanitation, and hygiene interventions, particularly during periods of high temperature and heavy rainfall.

Although significant associations between meteorological factors and childhood diarrhea were observed, the surveillance dataset did not include information on the specific biological agents responsible for diarrheal illness. Consequently, it was not possible to determine whether the observed climatic associations differed according to bacterial, viral, or parasitic pathogens, each of which may respond differently to temperature and rainfall. Therefore, the findings should be interpreted as reflecting overall childhood diarrhea rather than pathogen-specific transmission dynamics. Future studies integrating microbiological surveillance with meteorological data would improve understanding of pathogen-specific responses to climatic variability and support more targeted public health interventions [15,54].

Although this study identified significant associations between monthly meteorological variables and childhood diarrhea, climatic exposures may also influence delayed effects because of pathogen incubation periods, environmental persistence, and behavioural responses. Therefore, the observed associations should be interpreted as short-term associations rather than immediate causal effects. Because the available data were aggregated at the monthly level over a 12-month study period, lagged analyses and formal assessment of temporal autocorrelation were beyond the scope of this study. Therefore, the observed associations should be interpreted as short-term associations rather than immediate causal effects. Future studies using longer time-series data and distributed lag or other time-series models are warranted to better characterise the delayed effects of climatic variability on childhood diarrhea.

In addition to meteorological factors, younger child age was significantly associated with a higher prevalence of childhood diarrhea. Each additional month of age was associated with a 2% lower prevalence of childhood diarrhea, suggesting that younger children are more susceptible to diarrheal diseases, possibly because of their immature immune systems and increased vulnerability during the transition to complementary feeding, for example, those aged between 6 and 11 months begin to crawl and walk, which increases their chance of ingesting diarrheal pathogens from contaminated materials, as it has been observed elsewhere [55,56]. As children grow older, improved immune development, better hygiene practices, and reduced susceptibility to enteric pathogens may contribute to the observed decline in diarrhea prevalence. This finding is consistent with previous studies reporting a higher burden of diarrhea among younger children aged 6–23 months than among older children [55–57].

Moreover, we found that children whose mothers had a primary level of education had a lower prevalence of childhood diarrhea than those whose mothers had college or higher education. Although similar findings have been reported in some East African countries [57,58], this contrasts with the widely reported protective effect of higher maternal education on child health outcomes. One possible explanation is that mothers with higher educational attainment are more likely to be formally employed and therefore may spend less time providing direct childcare, relying instead on alternative caregivers [59]. Differences in healthcare-seeking behaviour may also contribute, as more educated mothers may be more likely to seek healthcare for childhood illnesses, increasing the likelihood of case detection in health facility-based surveillance. However, these explanations remain speculative because the present study did not collect detailed information on childcare practices, caregiver characteristics, household income, or other socio-behavioural factors that may influence childhood diarrhea. Therefore, this finding should be interpreted with caution and warrants further investigation using studies incorporating more comprehensive socioeconomic and behavioural data to better understand the underlying mechanisms [60,61].

### Limitations and strengths

The study has several limitations. First, the prevalence of childhood diarrhea may have been overestimated because the analysis was based on healthcare records, where some children could have presented with multiple concurrent illnesses. Second, the retrospective analysis of repeated cross-sectional surveillance data precludes causal inference. Third, lagged effects and temporal autocorrelation between meteorological variables and childhood diarrhea were not evaluated because the available data were aggregated at the monthly level over a relatively short study period. Finally, although handwashing practices, food preservation, and latrine type were assessed, they were not retained in the final model, while important determinants such as household income, nutritional status, population density, and detailed water quality measures were unavailable in the surveillance dataset, leaving the possibility of residual confounding. Despite these limitations, the study provides valuable evidence on seasonal patterns and short-term associations between meteorological factors and childhood diarrhea across diverse climatic zones in Tanzania by integrating standardised health surveillance (DHIS2) and meteorological (TMA) data. These findings may help inform public health interventions during periods of increased diarrheal risk.

## Conclusion and recommendations

The prevalence of childhood diarrhea among children under five was highest during the dry season in the unimodal regions, led by the Mbeya region, and highest during the rainy season in the bimodal regions, led by the Mwanza region. We found a positive relationship between an average monthly increase of 1 ^0^C in temperature, and a 1 mm increase in total monthly rainfall, with increasing prevalence of childhood diarrhea. These results highlight the need for public health interventions, including environmental conservation, protecting water contamination areas, preserving food, and training individuals on drinking treated water and hygiene practices.
