# Supplementary material for: Seasonal trends and short-term association between meteorological factors and diarrheal diseases among children under five in Tanzania
Source: PLoS One. 2026 Aug 28;21(8):e0357174. doi: 10.1371/journal.pone.0357174 (PMC13524289; doi:10.1371/journal.pone.0357174)
Supplement: Supplementary file 1 [file pone.0357174.s001.pdf]

Reviewer Comments

Seasonal Trends and Short-term Association between Weather Variables and Diarrheal Diseases among Under five Children in Tanzania.

General Comments

This manuscript has great public health contribution by exploring the impact of climatic factors on childhood diarrhea in Tanzania in areas with different rainfall patterns. The subject is extremely important, particularly given the growing effects of climate change on the health of children in Sub-Saharan Africa. Strengths of the study include the integration of meteorological data and health records, the application of multilevel analysis, and the inclusion of various climatic zones. However, there are some major issues related to methodology, analysis and presentation that need to be clarified and improved before the manuscript is consider for publication.

The manuscript need major revisions to improve clarity, methodological rigor, consistency of interpretation, and scientific writing.

Major Comments

1. Inconsistent in study design used and outcome variable definition

The manuscript describes the study as a "cross-sectional study" while also analyzing seasonal trends over a 12-month period using monthly climatic data. This creates confusion regarding the true design of the study used

Additionally, the outcome variable is described as "diarrhea illness occurring within two weeks preceding the survey" but the data were extracted retrospectively from DHIS2 records. The authors should clarify:

a. Whether this was repeated cross-sectional surveillance data,
b. How participants were enrolled prospectively,
c. How the "two-week recall" was operationalized using data in health facility records.

So, the study design needs to write more accurate.

2. Selection bias and limited representativeness

The study included children who get care at only 10 selected hospitals. This may not represent the broader under-five population in Tanzania, especially community cases that do not reach healthcare facilities.

The manuscript should discuss:

a. Potential healthcare-seeking bias,
b. Urban-rural representativeness,
c. Selection criteria for hospitals,

Whether findings can truly be generalized nationally.

3. Statistical Modeling

The authors mention using:

“multilevel logistic regression model with modified Poisson”

This description is statistically unclear because logistic regression and modified Poisson regression are different approaches.

The authors should clearly specify:

a. The exact model used,
b. The STATA command/package,
c. Whether robust standard errors were applied,
d. Why prevalence ratios (APR) is preferred over odds ratios.

In addition, the equations presented appear inconsistent with a binary outcome and resemble count models. This section needs substantial clarification.

4. Interpretation regarding age and diarrhea

There is a major contradiction in interpretation.

In results section: as children grow older the probability of having diarrhea reduces by 13%. However, in discussion section it says as child grow older the chance of having diarrhea is increases.

These statements directly contradict each other. The interpretation should be corrected throughout the manuscript.

5. Temporal Relationship between Climate Variables and Diarrhea

The study uses monthly rainfall and temperature averages, but diarrheal diseases often have lag effects. The authors should discuss:

    a. Why lagged analyses were not performed,
    b. Whether same month exposure is biologically appropriate,
    c. Potential temporal autocorrelation.

6. Unaddressed Important Confounders variables

Diarrhea has several determinants but in this study they were not included such as household income, nutritional status, population density, handwashing facility availability, VIP/Latrine utilization, seasonal food insecurity, water quality and amount (scarcity).

Minor Comments

7. Grammar and Language Issues

The manuscript contains numerous grammatical and typographical errors. Examples include:

“conditionsis” “modelbuilding” “there were no any diarrhea cases”

Professional English editing is recommended.

8. Table format is not followed standardized Table formats
   Presence of more vertical line that make the table condense.
9. Consistency of Terminology

The manuscript use different naming for Diarrhea: diarrhea, diarrheal diseases, childhood diarrhea, diarrhea prevalence. So better to use consistent terminology throughout the manuscript

10. Data Availability Statement

The data availability statement restricts access through institutional approval. The authors should ensure compliance with the journal’s data-sharing policy and explain whether de-identified datasets could be deposited in a repository.

7. Ethical Statement

The ethics section is appropriate, but since secondary data were used, clarify how written informed consent was obtained for this retrospectively types of study

Recommendation

Major Revision

The manuscript has scientific merit and addresses an important topic, but substantial revisions are needed in methodological clarity, interpretation of findings, statistical modeling, and language quality before publication consideration.
