# Supplementary material for: Seasonal trends and short-term association between meteorological factors and diarrheal diseases among children under five in Tanzania
Source: PLoS One. 2026 Aug 28;21(8):e0357174. doi: 10.1371/journal.pone.0357174 (PMC13524289; doi:10.1371/journal.pone.0357174)
Supplement: Supplementary file 2 [file pone.0357174.s003.pdf]

Manuscript Title: Seasonal Trends and Short-term Association Between Weather Variables and Diarrheal Diseases Among Under-five Children in Tanzania

Manuscript ID: PONE-D-25-34946

Dear Academic Editor and Reviewers,

We sincerely thank you for your careful evaluation of our manuscript and for your constructive comments and suggestions. We have carefully addressed all comments and revised the manuscript accordingly. The revisions have improved the clarity, methodological rigour, and presentation of the manuscript. Our point-by-point responses are provided below.

Response to Academic Editor

Comment 1: Funding Statement

Response: We thank the Academic Editor for this observation. Funding information has been removed from the Acknowledgements section of the manuscript. The Funding Statement remains unchanged and will appear only in the Funding Statement section of the online submission system. We have removed funding-related text from the Acknowledgements section.

Responses to Reviewer #1

Comment 1: Study design and outcome definition

Response: We thank the reviewer for this comment. We agree that describing the study simply as a "cross-sectional study" may create confusion because the analysis combined routinely collected health surveillance data over 12 months with corresponding meteorological data. We have revised the manuscript to clarify that the study was a retrospective analysis of repeated cross-sectional surveillance data collected through the ongoing Seq-Tanzania project and stored in DHIS2. Participants were not prospectively enrolled specifically for this study; rather, we analysed existing records collected during routine surveillance activities between May 2023 and April 2024.

We have also revised the description of the outcome variable to clarify that childhood diarrhea was identified during routine clinical assessments using the standardised Seq-Tanzania surveillance tool. During healthcare visits, trained healthcare workers interviewed parents or legal guardians and assessed diarrhea according to the World Health Organisation (WHO) case definition before recording the information in DHIS2. The previous wording referring to

"diarrhea occurring within two weeks preceding the survey" has therefore been removed because it did not accurately describe the data collection process.

Comment 2: Selection bias and representativeness

Response: Thank you for this important observation. We acknowledge that the study population was restricted to children attending 10 healthcare facilities participating in the Seq-Tanzania surveillance system and, therefore, may not fully represent all under-five children in Tanzania, particularly those who do not seek formal healthcare services.

We have revised the manuscript and clarify on the healthcare facility selection process and added a discussion on potential healthcare-seeking bias, urban-rural representativeness, and the limitations regarding national generalizability. The selected facilities were purposively chosen based on their participation in the Seq-Tanzania project, geographic distribution across major climatic zones, patient volume, and laboratory capacity. While the inclusion of facilities from both mainland Tanzania and Zanzibar and from regions with distinct unimodal and bimodal rainfall patterns improves climatic representativeness, the findings should be interpreted as representative of children seeking care within the surveillance network rather than the entire under-five population of Tanzania.

Comment 3: Statistical modelling

Response: We thank the reviewer for this comment. We agree that the previous description of the statistical model was inaccurate and could lead to confusion. The manuscript has been revised to clearly state that we used a multilevel mixed-effects Poisson regression model with a log link function and robust standard errors, implemented using the “meglm” command in STATA version 17, to estimate adjusted prevalence ratios (APR).

We have also clarified that prevalence ratios were preferred over odds ratios because childhood diarrhea was a common outcome in our study population (>10%), for which odds ratios may overestimate the strength of association. In addition, the statistical analysis section has been simplified to improve clarity. Unnecessary derivations and repetitive model descriptions were removed to improve readability while retaining sufficient methodological detail for reproducibility.

Comment 4: Age interpretation

Response: We thank the reviewer for noting this inconsistency. We agree that the interpretation in the Discussion section contradicts the findings presented in the Results. The manuscript has been revised to ensure consistency throughout. The Results section has been corrected to reflect the final model estimates, indicating that each additional month of age was

associated with a 2% lower prevalence of childhood diarrhea. The Discussion has also been revised accordingly to reflect this inverse association.

Comment 5: Lag effects

Response: We thank the reviewer for this comment. We agree that climatic factors may influence diarrheal diseases with delayed (lagged) effects. In this study, our objective was to assess short-term associations between monthly meteorological conditions and childhood diarrhea using routinely collected surveillance data. Because the available health and meteorological data were aggregated at the monthly level and the study was not designed to evaluate distributed lag effects, lagged analyses and temporal autocorrelation modelling were not assessed in the current study. We have acknowledged this as a limitation and expanded the discussion to clarify that future studies using longer time series and advanced time-series approaches, such as distributed lag models, are needed to better characterise delayed climatic effects on childhood diarrhea.

Comment 6: Confounders

Response: We thank the reviewer for this comment. We acknowledge that childhood diarrhea is influenced by several socioeconomic, environmental, and behavioural factors. Variables including household income, nutritional status, population density, and detailed measures of water quality and water scarcity were not available in the routine surveillance data collected through the Seq-Tanzania project. Handwashing practices, food preservation, and latrine type were available and were evaluated during model building; however, they were not retained in the final multivariable model because they were not significantly associated with childhood diarrhea and did not improve model fit. We have clarified these points in the Limitations section and acknowledged the potential for residual confounding.

Responses to Reviewer #2

Comment 1: Grammar

Response: We thank the reviewer for this observation. The manuscript has been thoroughly revised to correct grammatical, typographical, and formatting errors. Moreover, the entire manuscript has undergone careful English language editing to improve clarity, readability, and overall scientific presentation.

Comment 2: Table formatting

Response: We thank the reviewer for this suggestion. The formatting of all tables has been revised to conform to the journal's recommended table style. Unnecessary vertical lines have been removed, and the tables have been reformatted to improve readability and presentation.

Comment 3: Terminology

Response: We thank the reviewer for this suggestion. We have carefully revised the manuscript to ensure consistent use of terminology throughout.

Comment 4: Data Availability

Response: We thank the reviewer for this comment. The data analysed in this study were obtained from the Seq-Tanzania project and are subject to data ownership and sharing agreements. The authors are not authorised to deposit the dataset in a public repository. However, de-identified data may be made available upon request and with approval from the Seq-Tanzania project and the relevant institutional authorities, in accordance with applicable data-sharing policies. We have revised the Data Availability Statement to clarify this.

Comment 5: Ethics

Response: We thank the reviewer for this comment. We have revised the Ethics Statement to clarify that this study involved a secondary analysis of routinely collected surveillance data from the Seq-Tanzania project. Written informed consent and assent (where applicable) were obtained from participants or the parents/legal guardians of children during the original Seq-Tanzania data collection. The present study used de-identified data and did not involve direct participant contact or additional data collection.

We once again sincerely thank the Academic Editor and the reviewers for their constructive comments and insightful suggestions. We believe that the revisions made have strengthened the manuscript, and we hope that the revised version is now suitable for publication.

Yours sincerely,

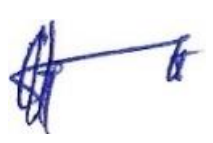

Gerald P. Mwing’a

Corresponding author

Email: gerald.mwinga@nimr.or.tz
