# Supplementary material for: Seasonal trends and short-term association between meteorological factors and diarrheal diseases among children under five in Tanzania
Source: PLoS One. 2026 Aug 28;21(8):e0357174. doi: 10.1371/journal.pone.0357174 (PMC13524289; doi:10.1371/journal.pone.0357174)
Supplement: Peer review history

# Peer review: Decision Letter 0

**DOI:** 10.1371/journal.pone.0357174.r001

**Authors:** James Colborn

27 May 2026

Dear Dr. Mwing'a,

Please submit your revised manuscript by Jul 11 2026 11:59PM. If you will need more time than this to complete your revisions, please reply to this message or contact the journal office at plosone@plos.org. When you're ready to submit your revision, log on to https://www.editorialmanager.com/pone/ and select the 'Submissions Needing Revision' folder to locate your manuscript file.

- A letter that responds to each point raised by the academic editor and reviewer(s). You should upload this letter as a separate file labeled 'Response to Reviewers'.
- A marked-up copy of your manuscript that highlights changes made to the original version. You should upload this as a separate file labeled 'Revised Manuscript with Track Changes'.
- An unmarked version of your revised paper without tracked changes. You should upload this as a separate file labeled 'Manuscript'.

If you would like to make changes to your financial disclosure, please include your updated statement in your cover letter. Guidelines for resubmitting your figure files are available below the reviewer comments at the end of this letter.

If applicable, we recommend that you deposit your laboratory protocols in protocols.io to enhance the reproducibility of your results. Protocols.io assigns your protocol its own identifier (DOI) so that it can be cited independently in the future. For instructions see: https://journals.plos.org/plosone/s/submission-guidelines#loc-laboratory-protocols. Additionally, PLOS ONE offers an option for publishing peer-reviewed Lab Protocol articles, which describe protocols hosted on protocols.io. Read more information on sharing protocols at https://plos.org/protocols?utm_medium=editorial-email&utm_source=authorletters&utm_campaign=protocols.

As the corresponding author, your ORCID iD is verified in the submission system and will appear in the published article. PLOS supports the use of ORCID, and we encourage all coauthors to register for an ORCID iD and use it as well. Please encourage your coauthors to verify their ORCID iD within the submission system before final acceptance, as unverified ORCID iDs will not appear in the published article. *Only*  the individual author can complete the verification step; PLOS staff *cannot*  verify ORCID iDs on behalf of authors.

We look forward to receiving your revised manuscript.

Kind regards,

James Colborn

Academic Editor

PLOS One

Journal Requirements:

When submitting your revision, we need you to address these additional requirements.

1. Please ensure that your manuscript meets PLOS ONE's style requirements, including those for file naming. The PLOS ONE style templates can be found at

https://journals.plos.org/plosone/s/file?id=wjVg/PLOSOne_formatting_sample_main_body.pdf and

https://journals.plos.org/plosone/s/file?id=ba62/PLOSOne_formatting_sample_title_authors_affiliations.pdf

2. Thank you for stating the following in the Acknowledgments Section of your manuscript:

“This study was funded by the Danish International Development Agency (DANIDA) through the Seq-Tanzania project (Grant No. 20-12-TAN) to cover the costs of data collection. The funders had no role in study design, data collection and analysis, decision to publish, or preparation of the manuscript.”

We note that you have provided funding information that is not currently declared in your Funding Statement. However, funding information should not appear in the Acknowledgments section or other areas of your manuscript. We will only publish funding information present in the Funding Statement section of the online submission form.

Please remove any funding-related text from the manuscript and let us know how you would like to update your Funding Statement. Currently, your Funding Statement reads as follows:

“This study was funded by the Danish International Development Agency (DANIDA) through the Seq-Tanzania project (Grant No. 20-12-TAN) to cover the costs of data collection. The funders had no role in study design, data collection and analysis, decision to publish, or preparation of the manuscript.”

Please include your amended statements within your cover letter; we will change the online submission form on your behalf.

3. In the online submission form you indicate that your data is not available for proprietary reasons and have provided a contact point for accessing this data. Please note that your current contact point is a co-author on this manuscript. According to our Data Policy, the contact point must not be an author on the manuscript and must be an institutional contact, ideally not an individual. Please revise your data statement to a non-author institutional point of contact, such as a data access or ethics committee, and send this to us via return email. Please also include contact information for the third party organization, and please include the full citation of where the data can be found.

4. If the reviewer comments include a recommendation to cite specific previously published works, please review and evaluate these publications to determine whether they are relevant and should be cited. There is no requirement to cite these works unless the editor has indicated otherwise.

[Note: HTML markup is below. Please do not edit.]

Reviewers' comments:

Reviewer's Responses to Questions

**Comments to the Author**

1. Is the manuscript technically sound, and do the data support the conclusions?

Reviewer #1: Yes

Reviewer #2: Yes

**********

2. Has the statistical analysis been performed appropriately and rigorously?

Reviewer #1: Yes

Reviewer #2: No

**********

3. Have the authors made all data underlying the findings in their manuscript fully available?

The PLOS Data policy

Reviewer #1: No

Reviewer #2: Yes

**********

4. Is the manuscript presented in an intelligible fashion and written in standard English?

Reviewer #1: Yes

Reviewer #2: No

**********

Reviewer #1: Review Report: PONE-D-25-34946

Full Title: Seasonal trends and short-term association between weather variables and diarrheal diseases among under-five children in Tanzania.

General Comment: This study investigates the seasonal prevalence and short-term associations between weather variables (temperature and rainfall) and diarrheal diseases among under-five children in seven regions of Tanzania. Utilizing a cross-sectional design and multilevel logistic regression, the authors found that childhood diarrhea prevalence varied significantly by season and rainfall pattern (dry season in unimodal regions, wet season in bimodal regions). Both increased temperature and rainfall were significantly associated with increased diarrhea occurrence. The study provides valuable insights into the climatic drivers of childhood diarrhea in a diverse geographical context.

Specific comments

1. Title: The Short Title "Seasonal trends and factors associated with childhood diarrhea in Tanzania" is less informative than the Full Title as it omits the crucial mention of "weather variables" as the primary factors investigated. The Short Title should be revised to more accurately reflect the core focus on weather variables, perhaps "Seasonal trends and weather factors associated with childhood diarrhea in Tanzania."

2. Abstract: The abstract is comprehensive, covering all essential components (background, objective, methodology, key results, and conclusion) clearly and concisely. It provides sufficient information for a reader to understand the study's scope and main findings.

3. Background: While informative, it could be strengthened by providing more specific regional or continental context regarding known diarrhea patterns or climatic influences in East Africa/Sub-Saharan Africa. This would further ground the study within the broader regional epidemiology. The authors should briefly incorporate a few sentences summarizing key findings or trends from other East African or Sub-Saharan African countries regarding the specific impact of temperature and rainfall on childhood diarrhea, or known regional disparities in water/sanitation infrastructure within Tanzania.

4. Methodology

Cross-sectional Design: As acknowledged by the authors, this design limits causal inference. While unavoidable for this study, it's a fundamental limitation.

Aggregated Climatic Data: Using regional average weather data for individual-level analysis introduces potential ecological fallacy. Individual children's exposure might vary significantly from regional averages.

Reliance on Secondary Data (Diarrhea Definition): The potential for overestimation of diarrhea prevalence due to reliance on medical records (where other conditions might inflate diagnoses) is a valid concern raised by the authors themselves.

Missing Biological Agents: The most significant methodological gap is the absence of data on specific biological agents (bacteria, viruses, parasites) causing diarrhea. Different pathogens have distinct environmental sensitivities and seasonal patterns. Without this information, the observed associations between climate and diarrhea are a black box, limiting the depth of mechanistic understanding and the specificity of interventions. The authors should more explicitly address how the lack of biological agent data impacts the interpretation of the climatic associations and the specificity of recommendations. This is a critical point for future research.

5. Results

Presents clear, quantified findings for seasonal prevalence and the associations with temperature and rainfall. The use of tables and figures effectively conveys the data.

The finding regarding mother's education level (lower education associated with lower diarrhea prevalence) is presented as "surprising" and explained speculatively. While the authors acknowledge this, it suggests potential unmeasured confounders or biases that might not have been fully captured or adjusted for. The authors should reiterate in the discussion that the finding on mother's education requires further investigation with more detailed socio-behavioral data to understand the underlying mechanisms.

6. Discussion

As noted above, the discussion could be strengthened by explicitly addressing how the lack of biological agent data limits the mechanistic understanding of the climatic associations and the specificity of public health recommendations.

The authors should add a paragraph or two to the discussion specifically addressing the implications of not having biological agent data, and how this impacts the interpretation of seasonal and climatic trends.

7. Conclusion

It’s directly supporting the main findings of the study and translates them into actionable public health recommendations. It is concise and impactful.

8. Recommendation for Publication

The article presents a well-conducted study with robust statistical analysis and valuable findings relevant to public health in Tanzania. The authors have demonstrated a good understanding of their data and its limitations. The concerns raised are primarily about enhancing the depth of interpretation and acknowledging specific data gaps, rather than fundamental flaws in the study's execution or analysis.

Therefore, I recommend Minor Revision. The authors should address the specific points raised, particularly regarding the "Short Title," the background context, and a more explicit discussion of the implications of not having biological agent data. Once these minor revisions are addressed, the manuscript should be acceptable for publication.

Reviewer #2: Reviewer Comments

Seasonal Trends and Short-term Association between Weather Variables and Diarrheal Diseases among Under five Children in Tanzania.

General Comments

This manuscript has great public health contribution by exploring the impact of climatic factors on childhood diarrhea in Tanzania in areas with different rainfall patterns. The subject is extremely important, particularly given the growing effects of climate change on the health of children in Sub-Saharan Africa. Strengths of the study include the integration of meteorological data and health records, the application of multilevel analysis, and the inclusion of various climatic zones. However, there are some major issues related to methodology, analysis and presentation that need to be clarified and improved before the manuscript is consider for publication.

The manuscript need major revisions to improve clarity, methodological rigor, consistency of interpretation, and scientific writing.

Major Comments

1. Inconsistent in study design used and outcome variable definition

The manuscript describes the study as a “cross-sectional study” while also analyzing seasonal trends over a 12-month period using monthly climatic data. This creates confusion regarding the true design of the study used

Additionally, the outcome variable is described as “diarrhea illness occurring within two weeks preceding the survey” but the data were extracted retrospectively from DHIS2 records. The authors should clarify:

a. Whether this was repeated cross-sectional surveillance data,

b. How participants were enrolled prospectively,

c. How the “two-week recall” was operationalized using data in health facility records.

So, the study design needs to write more accurate.

2. Selection bias and limited representativeness

The study included children who get care at only 10 selected hospitals. This may not represent the broader under-five population in Tanzania, especially community cases that do not reach healthcare facilities.

The manuscript should discuss:

a. Potential healthcare-seeking bias,

b. Urban-rural representativeness,

c. Selection criteria for hospitals,

Whether findings can truly be generalized nationally.

3. Statistical Modeling

The authors mention using:

“multilevel logistic regression model with modified Poisson”

This description is statistically unclear because logistic regression and modified Poisson regression are different approaches.

The authors should clearly specify:

a. The exact model used,

b. The STATA command/package,

c. Whether robust standard errors were applied,

d. Why prevalence ratios (APR) is preferred over odds ratios.

In addition, the equations presented appear inconsistent with a binary outcome and resemble count models. This section needs substantial clarification.

4. Interpretation regarding age and diarrhea

There is a major contradiction in interpretation.

In results section: as children grow older the probability of having diarrhea reduces by 13%. However, in discussion section it says as child grow older the chance of having diarrhea is increases.

These statements directly contradict each other. The interpretation should be corrected throughout the manuscript.

5. Temporal Relationship between Climate Variables and Diarrhea

The study uses monthly rainfall and temperature averages, but diarrheal diseases often have lag effects. The authors should discuss:

a. Why lagged analyses were not performed,

b. Whether same month exposure is biologically appropriate,

c. Potential temporal autocorrelation.

6. Unaddressed Important Confounders variables

Diarrhea has several determinants but in this study they were not included such as household income, nutritional status, population density, handwashing facility availability, VIP/Latrine utilization, seasonal food insecurity, water quality and amount (scarcity).

Minor Comments

7. Grammar and Language Issues

The manuscript contains numerous grammatical and typographical errors. Examples include:

“conditionsis” “modelbuilding” “there were no any diarrhea cases”

Professional English editing is recommended.

8. Table format is not followed standardized Table formats

Presence of more vertical line that make the table condense.

9. Consistency of Terminology

The manuscript use different naming for Diarrhea: diarrhea, diarrheal diseases, childhood diarrhea, diarrhea prevalence. So better to use consistent terminology throughout the manuscript

10. Data Availability Statement

The data availability statement restricts access through institutional approval. The authors should ensure compliance with the journal’s data-sharing policy and explain whether de-identified datasets could be deposited in a repository.

7. Ethical Statement

The ethics section is appropriate, but since secondary data were used, clarify how written informed consent was obtained for this retrospectively types of study

Recommendation

Major Revision

The manuscript has scientific merit and addresses an important topic, but substantial revisions are needed in methodological clarity, interpretation of findings, statistical modeling, and language quality before publication consideration.

**********

what does this mean?). If published, this will include your full peer review and any attached files.

If you choose “no”, your identity will remain anonymous but your review may still be made public.

**Do you want your identity to be public for this peer review?**  For information about this choice, including consent withdrawal, please see our Privacy Policy

Reviewer #1: No

Reviewer #2: No

**********

[NOTE: If reviewer comments were submitted as an attachment file, they will be attached to this email and accessible via the submission site. Please log into your account, locate the manuscript record, and check for the action link "View Attachments". If this link does not appear, there are no attachment files.]

To ensure your figures meet our technical requirements, please review our figure guidelines: https://journals.plos.org/plosone/s/figures

You may also use PLOS’s free figure tool, NAAS, to help you prepare publication quality figures: https://journals.plos.org/plosone/s/figures#loc-tools-for-figure-preparation.

NAAS will assess whether your figures meet our technical requirements by comparing each figure against our figure specifications.

Attachment Submitted filename: Reviewer Comments. DD.pdf

# Author comment: Author response to Decision Letter 1

**DOI:** 10.1371/journal.pone.0357174.r002

9 Jul 2026

We sincerely thank the Academic Editor and the reviewers for their careful evaluation and constructive comments. We have thoroughly revised the manuscript in response to all comments. Specifically, we clarified the study design, revised the Methods section (including the data sources and statistical analysis), updated the Results and Discussion sections, strengthened the interpretation of the findings, addressed the limitations regarding lag effects, biological agent data, and potential confounding, standardized terminology throughout the manuscript, revised the Data Availability and Ethics Statements, reformatted the tables, and corrected grammatical and typographical errors. A detailed point-by-point response to each comment has been provided in the uploaded "Response to Reviewers" document.

Attachment Submitted filename: Response to Reviewers.pdf

# Peer review: Decision Letter 1

**DOI:** 10.1371/journal.pone.0357174.r003

**Authors:** James Colborn

13 Aug 2026

Seasonal Trends and Short-term Association Between Meteorological Factors and Diarrheal Diseases Among Children Under Five in Tanzania

PONE-D-25-34946R1

Dear Dr. Mwing'a,

We’re pleased to inform you that your manuscript has been judged scientifically suitable for publication and will be formally accepted for publication once it meets all outstanding technical requirements.

Within one week, you’ll receive an e-mail detailing the required amendments. When these have been addressed, you’ll receive a formal acceptance letter and your manuscript will be scheduled for publication.

An invoice will be generated when your article is formally accepted. Please note, if your institution has a publishing partnership with PLOS and your article meets the relevant criteria, all or part of your publication costs will be covered. Please make sure your user information is up-to-date by logging into Editorial Manager at Editorial Manager® and clicking the ‘Update My Information' link at the top of the page. For questions related to billing, please contact billing support.

If your institution or institutions have a press office, please notify them about your upcoming paper to help maximize its impact. If they’ll be preparing press materials, please inform our press team as soon as possible -- no later than 48 hours after receiving the formal acceptance. Your manuscript will remain under strict press embargo until 2 pm Eastern Time on the date of publication. For more information, please contact onepress@plos.org.

Kind regards,

James Colborn

Academic Editor

PLOS One

Additional Editor Comments (optional):

Reviewers' comments:

Reviewer's Responses to Questions

**Comments to the Author**

Reviewer #1: All comments have been addressed

Reviewer #2: All comments have been addressed

**********

2. Is the manuscript technically sound, and do the data support the conclusions?

Reviewer #1: Yes

Reviewer #2: Yes

**********

3. Has the statistical analysis been performed appropriately and rigorously?

Reviewer #1: Yes

Reviewer #2: Yes

**********

4. Have the authors made all data underlying the findings in their manuscript fully available?

The PLOS Data policy

Reviewer #1: Yes

Reviewer #2: Yes

**********

5. Is the manuscript presented in an intelligible fashion and written in standard English?

Reviewer #1: Yes

Reviewer #2: Yes

**********

Reviewer #1: Comments on the Revised Manuscript PONE-D-25-34946R1

Manuscript Title: Seasonal Trends and Short-term Association Between Weather Meteorological Factors and Diarrheal Diseases Among Children Under Five in Tanzania

I appreciate the opportunity to review the revised version of this manuscript.

The authors have carefully considered the comments raised during the initial round of my review and have provided detailed point-by-point responses. The revised manuscript has been substantially improved in terms of methodological clarity, scientific presentation, and interpretation of the findings.

The major concerns identified during the first review have been satisfactorily addressed.

Specifically :

- The study design has been clearly redefined as a retrospective analysis of repeated cross-sectional surveillance data, eliminating the ambiguity present in the original version;

- The description of the study population, healthcare facility selection, and case definition has been considerably strengthened, allowing readers to better understand the scope and representativeness of the study;

- The statistical analysis has been appropriately revised. The use of a multilevel mixed-effects Poisson regression model with robust standard errors is now correctly described and adequately justified;

- The inconsistency previously identified between the Results and the Discussion regarding the effect of age has been corrected;

- The Discussion now provides a more balanced interpretation of the findings and appropriately acknowledges important limitations, including residual confounding, healthcare-seeking bias, limited representativeness, and the absence of lag-effect analyses;

- The Data Availability Statement and Ethics Statement have been clarified in accordance with the journal's requirements;

- The manuscript has also benefited from substantial improvements in language, terminology, and overall readability.

Although some limitations remain, they are largely inherent to the study design and the available surveillance data rather than deficiencies of the analysis itself. These limitations are transparently acknowledged by the authors and do not substantially weaken the validity of the study's conclusions.

The manuscript provides useful evidence regarding the relationship between meteorological factors and childhood diarrhoea in Tanzania and contributes valuable information for disease surveillance and public health planning.

Overall, I am satisfied that the authors have adequately addressed to my comments.

Recommendation to the Editor: Accept for publication.

I congratulate the authors on the considerable improvements made during the revision process and have no further major comments.

Reviewer #2: Still, the manuscript needs some grammar editing and avoiding unnecessary information like Childhood Diarrhea remains one of the leading causes of morbidity and mortality among children under five in Tanzania. In case better to avoid "one of" and Table format is still needs revision to avoid unnecessary horizontal and vertical lines.

**********

what does this mean?). If published, this will include your full peer review and any attached files.

If you choose “no”, your identity will remain anonymous but your review may still be made public.

**Do you want your identity to be public for this peer review?**  For information about this choice, including consent withdrawal, please see our Privacy Policy

Reviewer #1: No

Reviewer #2: No

**********

# Editor report: Acceptance letter

**DOI:** 10.1371/journal.pone.0357174.r004

**Authors:** James Colborn

PONE-D-25-34946R1

PLOS One

Dear Dr. Mwing'a,

I'm pleased to inform you that your manuscript has been deemed suitable for publication in PLOS One. Congratulations! Your manuscript is now being handed over to our production team.

At this stage, our production department will prepare your paper for publication. This includes ensuring the following:

* All references, tables, and figures are properly cited

* All relevant supporting information is included in the manuscript submission,

* There are no issues that prevent the paper from being properly typeset

You will receive further instructions from the production team, including instructions on how to review your proof when it is ready. Please keep in mind that we are working through a large volume of accepted articles, so please give us a few days to review your paper and let you know the next and final steps.

Lastly, if your institution or institutions have a press office, please let them know about your upcoming paper now to help maximize its impact. If they'll be preparing press materials, please inform our press team within the next 48 hours. Your manuscript will remain under strict press embargo until 2 pm Eastern Time on the date of publication. For more information, please contact onepress@plos.org.

You will receive an invoice from PLOS for your publication fee after your manuscript has reached the completed accept phase. If you receive an email requesting payment before acceptance or for any other service, this may be a phishing scheme. Learn how to identify phishing emails and protect your accounts at https://explore.plos.org/phishing.

If we can help with anything else, please email us at customercare@plos.org.

Thank you for submitting your work to PLOS One and supporting open access.

Kind regards,

PLOS ONE Editorial Office Staff

on behalf of

Dr. James Colborn

Academic Editor

PLOS One
